# INDECISIVENESS ABOUT LONG-TERM CARE AMONG OLDER ADULTS

**DOI:** 10.1093/geroni/igad104.2637

**Published:** 2023-12-21

**Authors:** Allie Schierer, Amber Miller-Winder, Raven Relerford, Charles Olvera, Vanessa Ramirez-Zohfeld, Alaine Murawski, Lee A Lindquist

**Affiliations:** Northwestern University, Chicago, Illinois, United States; Northwestern University, Chicago, Illinois, United States; Northwestern University, Chicago, Illinois, United States; Northwestern University, Chicago, Illinois, United States; Northwestern University, Chicago, Illinois, United States; Northwestern University, Chicago, Illinois, United States; Northwestern University, Chicago, Illinois, United States

## Abstract

As cognition and function worsen, older adults often make decisions about living in long term care (LTC) or accepting support in the home. We are longitudinally studying LTC decision-making among a cohort of older adults (65+), who have viewed PlanYourLifespan.org (PYL), a proven-effective LTC planning tool. Subjects were surveyed at baseline (BL), administered PYL, then followed with surveys every 6 months. Participants were asked about LTC decisions in the event of worsening cognition and to describe those decisions. Responses were analyzed using a mixed-methods approach with open-ended responses coded using constant comparative analysis. Of the 293 subjects, mean age was 73 years, 72.7% (213) female, 40.4% (118) under-represented minority. Between baseline-18 months, 66.5% of subjects changed their LTC plans (28.7% one change, 24.23% two changes, 9.2% three changes) At 1 and 6 mos., the proportion of respondents who changed their decision from their prior timepoint was 44%. By 12 mos., this proportion has declined to 39%, and by 18 mos., the proportion further decreased to 34%. Higher indecision (Yes-No-Yes-No) was seen with changes in family caregivers, timing around COVID-19, and personal health. Decision permanence increased at 12-and 18-month time points. LTC decision-making fluctuates; however, over time, the decision-making progresses from circling to more permanence. Understanding LTC decision making is longitudinally important to understand and should be revisited every 6-12 months in clinical practice.

